# Predicting skip metastasis in lateral lymph nodes of papillary thyroid carcinoma based on clinical and ultrasound features

**DOI:** 10.3389/fendo.2023.1151505

**Published:** 2023-05-09

**Authors:** Min Zhao, Xinyu Shi, Ziran Zou, Runze Wen, Yixing Lu, Jihui Li, Jinming Cao, Bin Zhang

**Affiliations:** ^1^ Department of Nuclear Medicine, The First Affiliated Hospital of Soochow University, Suzhou, China; ^2^ Department of General Surgery, The First Affiliated Hospital of Soochow University, Suzhou, China; ^3^ Department of Ultrasound, The First Affiliated Hospital of Soochow University, Suzhou, China; ^4^ State Key Laboratory of Radiation Medicine and Protection, Soochow University, Suzhou, China

**Keywords:** papillary thyroid cancer, skip metastasis, lateral lymph node metastasis, nomogram, factors

## Abstract

**Background:**

Skip metastasis in papillary thyroid cancer (PTC), defined as lateral lymph node metastasis (LLNM) without the involvement of central lymph node metastasis (CLNM), is generally unpredictable. Our study aimed to develop a model to predict skip metastasis by using clinicopathological and ultrasound factors of PTC.

**Methods:**

We retrospectively reviewed the medical records of patients who underwent total thyroidectomy and central lymph node dissection (CLND) plus lateral lymph node dissection (LLND) between January 2019 and December 2021 at the First Affiliated Hospital of Soochow University. Furthermore, univariate and multivariate analyses assessed the clinical and ultrasound risk factors. Receiver operating characteristic (ROC) curves were used to find the optimal cut-off values for age and dominant nodule diameter. Multivariate logistic regression analysis results were used to construct a nomogram and were validated internally.

**Results:**

In all patients, the skip metastasis rate was 15.4% (41/267). Skip metastasis was more frequently found in patients with a tumour size ≤10 mm (OR 0.439; P = 0.033), upper tumour location (OR 3.050; P=0.006) and fewer CLNDs (OR 0.870; P = 0.005). After analysing the clinical and ultrasound characteristics of the tumour, five factors were ultimately associated with lateral lymph node skip metastasis and were used to construct the model. These factors were an age >40 years, tumour diameter <9.1 mm, upper tumour location, non-smooth margin and extrathyroidal extension. The internally evaluated calibration curves indicated an excellent correlation between the projected and actual skip metastasis probability. The nomogram performed well in discrimination, with a concordance index of 0.797 (95% CI, 0.726 to 0.867).

**Conclusions:**

This study screened for predictors of skip metastasis in PTC and established a nomogram that effectively predicted the risk of potential skip metastasis in patients preoperatively. The method can predict and distinguish skip metastases in PTC in a simple and inexpensive manner, and it may have future therapeutic utility.

## Introduction

Papillary thyroid carcinoma is a prevalent endocrine malignancy. It accounts for 90% of thyroid cancer cases, and its incidence is increasing worldwide (1, 2). An abundance of previous studies had reported that cervical lymph node in PTC occurs in a stepwise manner. Generally, lymph node metastasis in PTC involves the central compartment, the ipsilateral lateral compartment, and the contralateral lateral compartment (3–6). However, LLNM without CLNM is also found in PTC; this unpredictable lymph node metastasis pattern is known as “skip metastasis” (7).

In clinical practice, ultrasound is often used for the preliminary examination of cervical lymph node metastasis. Ultrasound has been reported to have poor sensitivity but good specificity in the diagnosis of CLNM. The ultrasound specialist will evaluate the central and lateral cervical regions for suspicious lymph nodes before surgery. Because the presence of thyroid reduces the visualization of the interventricular lymph nodes, it is often easier to ignore LLNM when no CLNM is found. Standard primary surgery can significantly reduce patients’ risk of recurrence and distant metastasis, while secondary surgery may significantly increase the incidence of surgical risk and complications. However, preventive LLND for patients without LLNM will also increase surgical complications and medical costs to a certain extent (8–10). Therefore, it is very important to accurately evaluate the status of cervical lymph node metastasis before surgery to select a reasonable surgical method and plan an accurate range of dissection according to the condition.

It has become a major challenge for most thyroid surgeons to control localized regional recurrence (11). A precise preoperative assessment of skip metastasis aids in establishing the surgical window, lowering the risk of recurrence and reducing death rates. The present study aimed to investigate the incidence and clinicopathologic risk factors for skip metastasis. In addition, we established skip metastasis in patients with PTC based on preoperative thyroid ultrasound, laboratory examination and clinical characteristics.

## Materials and methods

### Patients

This retrospective analysis originally examined PTC patients who underwent total thyroidectomy with LLND plus CLND at the First Affiliated Hospital of Soochow University between January 2019 and December 2021. The pathology section of our hospital classified each case as PTC with LLNM. The skip metastatic and non-skip metastatic groups were created from each set. The exclusion criteria included (1) distant metastases already present or other cancers at the time of diagnosis, (2) neck surgery or radiation history at the time of diagnosis, and (3) limited information or an unknown clinicopathologic profile.

### Surgery treatment

All patients underwent total thyroidectomy with LLND plus CLND. This study included both therapeutic and preventive cases of LLND. Therapeutic LLND is performed when LLNM is diagnosed by preoperative ultrasound, CT, and/or FNA. In addition, based on the surgeon’s experience, LLND can be performed prophylactically if the patient has high risk factors. CLND was performed to remove all lymph nodes and fibro-fatty tissue from the medial border of the common carotid artery to the midline of the trachea, and from the hyoid bone to the thoracic inlet. The typical therapy for LLNM at our institution is modified LLND incorporating stages II–V with preservation of the spinal accessory nerve, internal jugular vein, and sternocleidomastoid muscle. Unless otherwise noted, level I dissection was not conducted routinely.

### Data collection

Basic information, laboratory examination, thyroid ultrasound and pathological factors were collected. Basic information included the patient’s sex, age at diagnosis and status of underlying disease (hypertension, diabetes, and hyperlipidaemia). The laboratory indices included thyroid-stimulating hormone (TSH), thyroglobulin antibodies (TgAb), and thyroglobulin (Tg). The characteristics of preoperative thyroid ultrasound of the largest tumour or the most suspicious dominant nodule included the following features: diameter, location, flexibility score, component, echogenicity, shape, margin, ratio of tall to wide, extrathyroidal extension, calcification, and vascularization. Multifocality, bilaterality, and extrathyroidal extension were also enrolled. Histopathologic factors analysed on postoperative pathological examination included maximum tumour size, maximum tumour location, multifocality, bilaterality, extrathyroidal extension (ETE), coexistence of nodular goitre, coexistence of Hashimoto’s thyroiditis, number of central/lateral dissected lymph nodes and number of lateral metastatic lymph nodes.

### Statistical analysis

All statistical analyses were performed with the SPSS 20.0 package (IBM SPSS Inc., Chicago, USA) and R software (ver. 4.1.3, Institute of Statistics and Mathematics, Vienna, Austria). The chi-square test and the independent t test were performed for categorical and continuous variables, respectively. Multivariate logistic regression analysis was performed for significant factors, and P<0.05 was considered to indicate that the differences were statistically significant. ROC curves were constructed to determine the optimal cut-off value. Based on the results of multiple logistic regression analysis, significant predictors were combined to develop a nomogram. The AUC values and calibration curves were used to examine the discriminatory power and degree of consistency of our prediction model.

## Results

### Patient characteristics

The study ultimately enrolled 267 patients. We found that skip metastasis occurred in 41 (15.4%) of these patients, and this phenomenon was not detected in the remaining patients. We summarize the demographic and pathological tumour characteristics of these patients in **Table 1**. A summary of the preoperative ultrasonographic characteristics of the tumours and laboratory tests of all patients is presented in **Table 2**.

**Table 1:** Comparison of the clinicopathological factors of skip metastasis and non-skip metastasis in PTC patients.

Variables	Skip metastasis		P value	Multivariate analysis	P value
	Absent (N=226)	Present (N=41)		OR (95% CI)	
Sex					
Female	135	28			
Male	91	13	0.384		
Age (year)					
< 55	202	31			
≥ 55	24	10	**0.022**	2.489(0.934-6.631)	0.068
Diameter of largest tumour (mm)					
≤ 10	67	24			
> 10	159	17	**<0.001**	2.276(1.067-4.852)	**0.033**
Location of largest tumour					
Non-upper	134	17			
Upper	92	24	**0.040**	3.050(1.380-6.740)	**0.006**
Multifocality					
Absent	83	21			
Present	143	20	0.080		
Bilaterality					
Absent	112	30			
Present	114	11	**0.005**	0.491(0.219-1.098)	0.083
Extrathyroidal extension					
Absent	165	30			
Microscopic	34	3			
Gross	27	8	0.222		
Hashimoto’s thyroiditis					
Absent	154	31			
Present	72	10	0.340		
Nodular goitre					
Absent	176	29			
Present	50	12	0.319		
CLND number	9.35 ± 5.681	5.83 ± 4.748	**<0.001**	0.870(0.789-0.960)	**0.005**
LLND number	24.54 ± 13.603	22.29 ± 11.858	0.321		
LLNM number	5.78 ± 4.189	3.80 ± 3.422	**0.005**	0.885(0.776-1.010)	0.07

Bold values indicate that P-value is significant.

**Table 2:** Comparison of the preoperative examination features of skip metastasis and non-skip metastasis in patients with PTC.

Variable	Skip metastasis		P value
	Absent (N=226)	Present (N=41)	
Sex			
Female	135	28	
Male	91	13	0.384
Age (year)	38.95 ± 10.946	43.05 ± 12.779	0.033
US-Multifocality			
Absent	135	21	
Present	91	20	0.389
US-Bilaterality			
Absent	165	34	
Present	61	7	0.242
US-reported dominant nodule			
Diameter	18.00 ± 10.239	14.34 ± 10.317	0.036
Location			
Non-upper	146	19	
Upper	80	22	0.035
Flexibility score			
1	0	0	
2	15	5	
3	97	15	
4	97	16	
5	17	5	0.365
Component			
Solid	201	33	
Cystic-solid	25	8	0.193
Echogenicity			
Hypoechoic	221	38	
Iso/hyperechoic	5	3	0.108
Shape			
Regular	115	15	
Irregular	111	26	0.126
Margin			
Smooth	145	15	
Non-smooth	81	26	0.001
Ratio of tall to wide			
<1	168	34	
≥1	58	7	0.323
Extrathyroidal extension			
Absent	183	27	
Present	43	14	0.038
Calcification			
Absent/macrocalcification	46	12	
Microcalcification	180	29	0.219
Vascularization			
Absent	40	4	
Present	186	37	0.257
Hashimoto’s thyroiditis			
Absent	157	31	
Present	69	10	0.464
Nodular goitre			
Absent	177	29	
Present	49	12	0.313
TSH			
Low	6	3	
Normal	189	31	
High	31	7	0.204
Tg			
Low	54	7	
Normal	135	30	
High	37	4	0.267
TgAb			
Positive	57	8	
Negative	169	33	0.554
BMI			
<25	123	15	
≥25	103	26	0.042
Hypertension			
Absent	196	33	
Present	30	8	0.330
Diabetes			
Absent	213	38	
Present	13	3	0.719
Hyperlipidaemia			
Absent	145	31	
Present	81	10	0.21

### Clinicopathological factors for skip metastasis

Using univariate analysis, we compared the clinicopathological factors of the groups with and without skip metastases. In the group with skipped metastases, the following patient characteristics were more prevalent: age ≥55 (P = 0.022), tumour size ≤10 mm (P < 0.001), upper location (P = 0.040), bilaterality (P = 0.005), fewer CLNDs (P<0.001) and fewer LLNMs (P = 0.005). Furthermore, there were no significant differences in sex, multifocality, extrathyroidal extension, Hashimoto’s thyroiditis, nodular goitre, or LLND number between the skip metastasis group and non-skip metastasis group (all P > 0.05). Additionally, multivariate analysis revealed that tumour size ≤10 mm (OR 2.276; 95% CI 1.067-4.852; P = 0.033), upper tumour location (OR 3.050; 95% CI 1.380–6.740; P= 0.006) and fewer CLNDs (OR 0.870; 95% CI 0.789–0.960; P = 0.005) were independent factors for skip metastasis, as shown in **Table 1**.

### Preoperative examination features for skip metastasis

We initially looked at the association between preoperative clinical and ultrasound characteristics and skip metastasis using a univariate analysis to better understand the indicators of skip metastasis. The significant risk factors were as follows: age (P=0.033), tumour diameter (P=0.036), upper tumour location (P=0.035), non-smooth margin (P=0.001), extrathyroidal extension (P=0.038) and BMI ≥25 (P=0.042) (**Table 2**).

**Figure 1:**
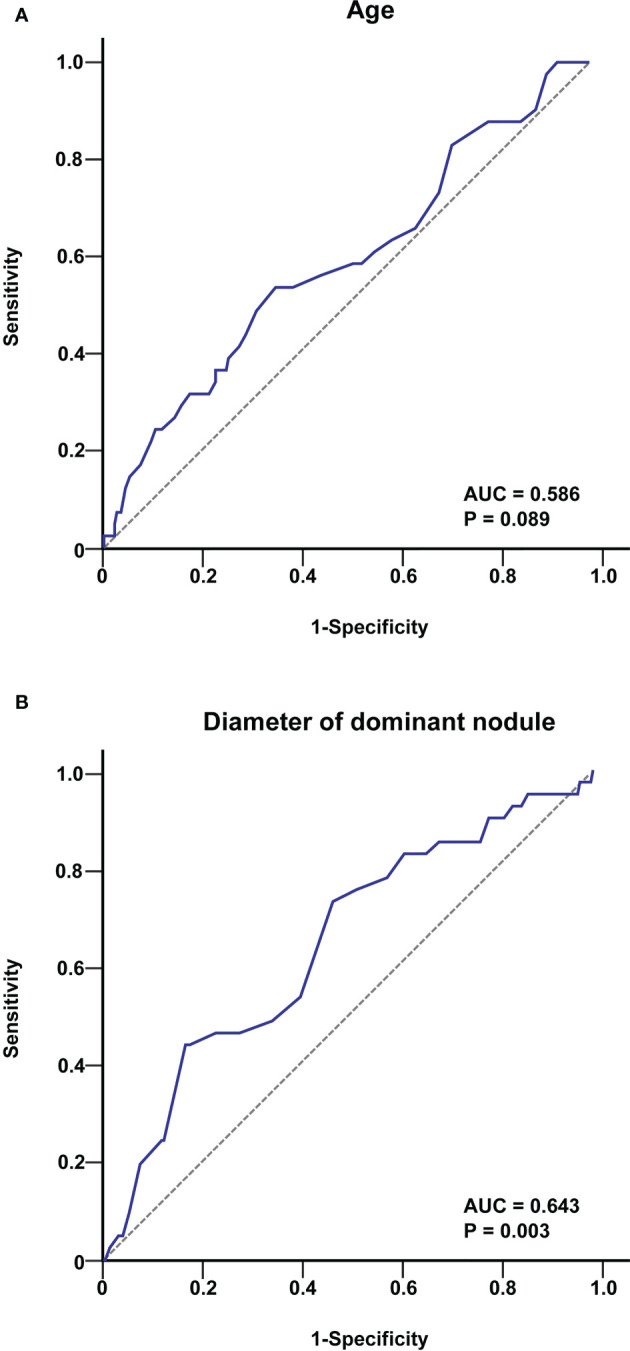
ROC curve analysis of age **(A)** and tumour diameter **(B)** for predicting skip metastasis in PTC patients. The cut-off value of age was 40 years, and that for the tumour diameter was 9.1 mm.

To further investigate the association between age and tumour diameter in PTC patients and the occurrence of skip metastasis, we created a ROC curve for 267 patients with PTC to establish the value of these parameters in predicting skip metastasis. The cut-off age was 40 years old, as shown in **Figure 1** [area under the curve (AUC) = 0.586, P = 0.089], and the tumour diameter was 9.1 mm (AUC = 0.643, P=0.003).

Further multivariate analysis indicated that tumour diameter <9.1 mm (OR 4.625; 95% CI 2.092–10.227; P<0.001), upper tumour location (OR 3.025; 95% CI 1.395–6.559; P=0.005), non-smooth margin (OR 4.104; 95% CI 1.874–8.987; P<0.001) and extrathyroidal extension (OR 2.251; 95% CI 1.014–4.996; P = 0.046) were independent predictors of skip metastasis in PTC (**Table 3**).

**Table 3:** Multivariate analysis of the predictive factors for skip metastasis in PTC patients.

Variable	OR (95% CI)	P value
Age (>40 years)	2.102(0.989-4.465)	0.053
BMI (≥25)	1.987(0.922-4.283)	0.080
US-reported dominant nodule		
Diameter (<9.1 mm)	4.625(2.092-10.227)	<0.001
Located in the upper pole	3.025(1.395-6.559)	0.005
Margin (Non-smooth)	4.104(1.874-8.987)	<0.001
Extrathyroidal extension	2.251(1.014-4.996)	0.046

### Construction of an individualized prediction model

**Figure 2:**
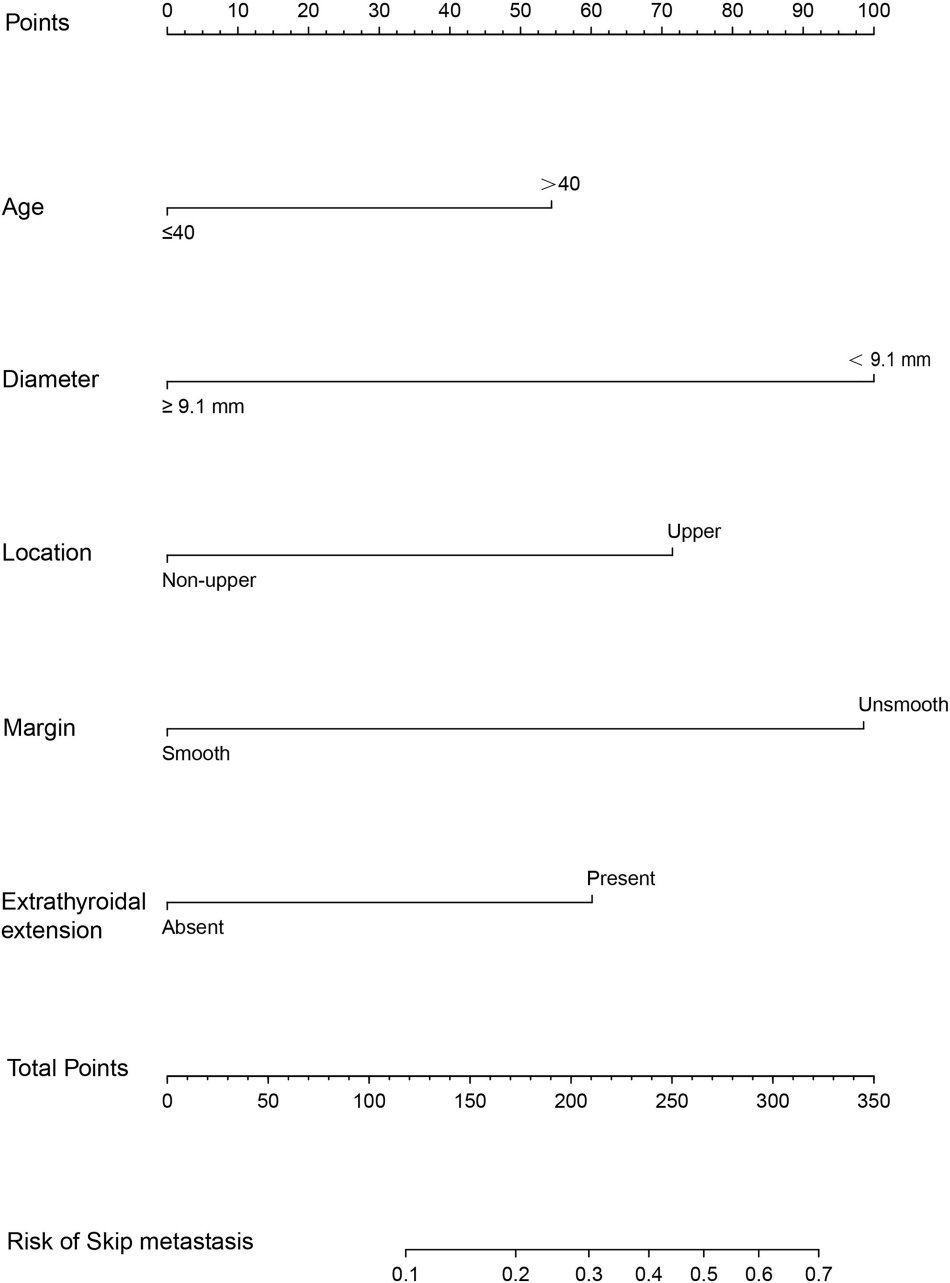
The nomogram for predicting the risk of skip metastasis in PTC patients.

**Figure 3:**
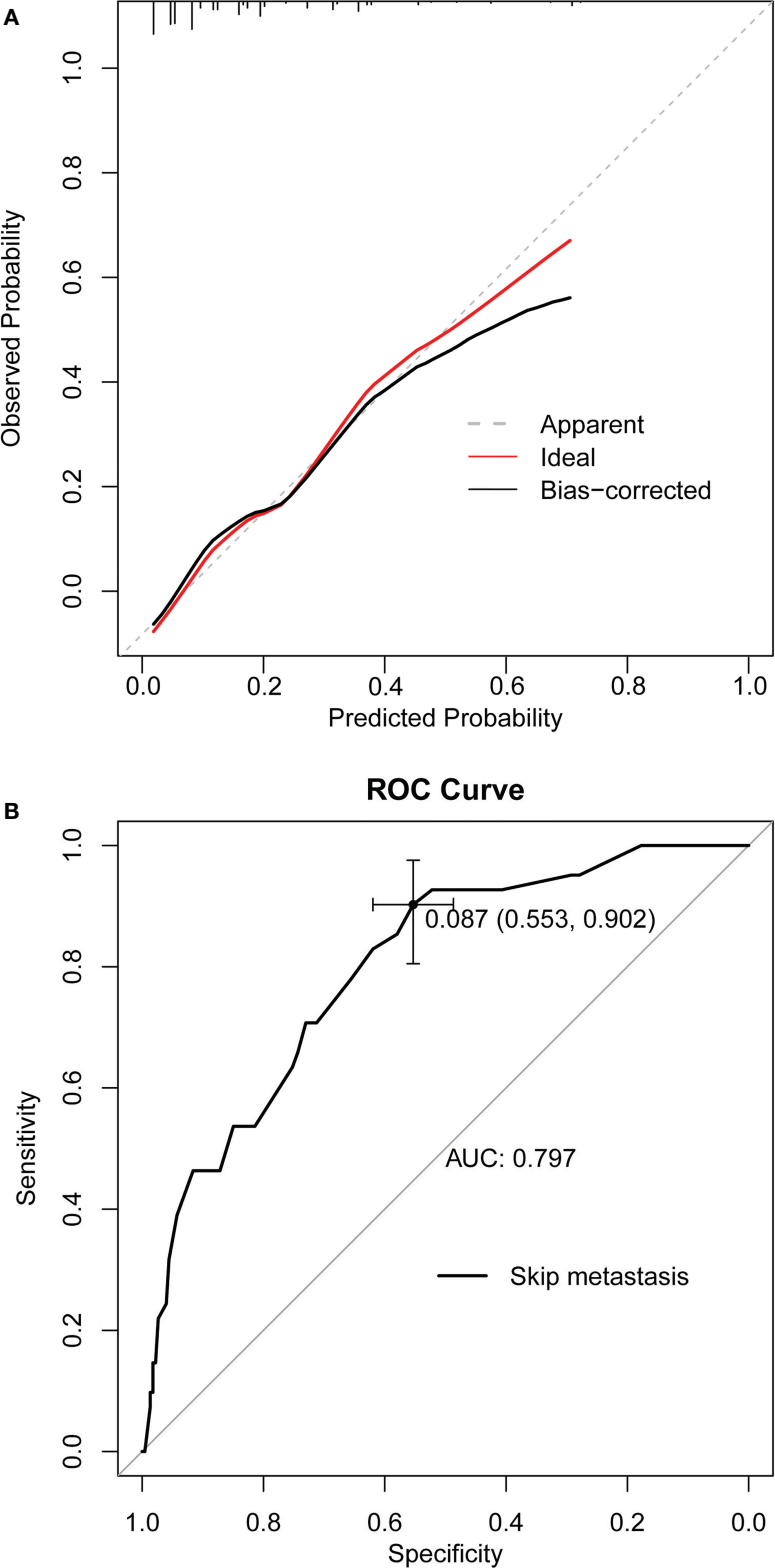
**(A)** Curves with internal validation for the nomogram. **(B)** ROC analysis of the nomogram. The ROC curve of nomograms for skip metastasis. The area under the ROC curve (AUC) was 0.797, 95% CI 0.726-0.867. ROC, receiver operating characteristic; AUC, area under curve.

A nomogram was developed for forecasting each individual’s probability of skip metastasis based on the independent characteristics assessed using multivariate analysis (**Figure 2**). The risk of each factor, including the diameter, location, and margin of the dominant nodule and extrathyroidal extension, was quantified in our prediction model based on the results of the multivariate analysis. As previously indicated, the univariate analysis revealed a difference in age between the skip metastasis and non-skip metastasis groups, and in the multivariate analysis, its P value was 0.053, which is close to 0.05. Therefore, we decided to include age as well. It was simple to calculate the estimated chance of skip metastases in LLNM patients by combining the scores for each variable and then drawing a straight line. The likelihood of skip metastases was often higher in the patients with higher total scores. The predicted chance of skip metastases and the actual observed skipped metastases were in good agreement, according to the internally confirmed calibration curves (**Figure 3A**). As shown in **Figure 3B**, the performance of the nomogram was validated internally, with an AUC of 0.797 (95% CI 0.726-0.867).

## Discussion

When the central lymph node is found to be negative by intraoperative pathology, no further LLND will be performed unless preoperative ultrasound-guided fine-needle aspiration biopsy (FNAB) and imaging demonstrate LLNM (12). However, LLNM *via* preoperative examination is proven to have a significant false-negative rate (4, 13), and the accuracy greatly depends on the pathologists’ and ultrasound operators’ experience (14). Underestimating skip metastases in PTC will result in insufficient lymph node dissection during surgery, which will ultimately have a negative impact on the prognosis of PTC patients. Therefore, it is crucial for surgeons to perform an accurate preoperative evaluation and prognosis of cervical lymph nodes.

The skip metastasis rate in our study with a large sample was 15.4% (41/267), which is in the range of 0.6% to 37.5% reported in previous studies (15–22). The greater skip metastatic rate shows that the occurrence of skip metastasis in our clinical work has not gone unnoticed.

The clinicopathological characteristics and risk factors for skip lateral lymph node metastasis in PTC patients were examined in this retrospective analysis. In the univariate and multivariate analyses, the rate of skip metastasis was significantly higher in patients with a tumour size ≤10 mm (P = 0.033), upper tumour location (P = 0.006) and fewer CLNDs (P = 0.005). Previously, many studies (18, 20, 23) have reported that skip lymph node metastasis is associated with tumour size, and skip metastasis is often found to be more common in PTC patients with a tumour size ≤1 cm. Several of these studies (15, 24, 25) found that the location of the tumour in the upper pole is one of the independent risk factors for the development of skip metastasis in patients with PTC. This could be because the upper pole of the thyroid lobe has a distinct lymphatic drainage system from that of the remainder of the thyroid lobe. Lymphatic flow through the superior thyroid artery is more likely to carry PTC cells from the upper area to the lateral lymph nodes. In addition, we discovered that the probability of skip metastasis was negatively correlated with the number of lymph nodes removed in the central neck (P = 0.005). A small number of central lymph nodes that have been removed may cause the probability of skip metastases to be overestimated (26, 27). A total CLND may eradicate all CLNMs and reduce the likelihood of false-positive skip metastasis detection.

The predicted variables associated with skip metastases in PTC were then investigated. Age, tumour diameter, upper tumour site, non-smooth margin, extrathyroidal extension, and BMI ≥25 were all associated with favourable outcomes in the univariate analysis (all P<0.05). Age and tumour diameter have been previously reported as risk factors for skip metastasis, so their relationship with skip rate was further investigated. To identify these parameters’ critical levels for predicting skip metastases in 267 PTC patients, we built ROC curves. According to our findings, the tumour diameter was 9.1 mm, and the cut-off age in PTC for skip metastasis was 40 years old. Age > 40 and tumour diameter < 9.1 mm are therefore thought to be used as thresholds for skip metastasis. Further multivariate analysis indicated that tumour diameter <9.1 mm, upper tumour location, non-smooth margin and extrathyroidal extension were independent predictors of skip metastasis in PTC. Furthermore, Zhao et al. (20) discovered by multivariate analysis that an age > 45 years was an independent risk factor for skip metastasis (OR 4.37; 95% CI 1.14-16.66; P = 0.031). Hu et al. (27) discovered that an older age (OR 2.63; 95% CI 1.34-5.04, P = 0.004) was an independent risk factor for skip metastasis. In our multifactorial analysis, the p value for an age >40 years was 0.053, which is close to 0.05. Therefore, we decided to include age as well.

A predictive nomogram was constructed based on the above significant factors of preoperative clinical and ultrasound features associated with PTC skip metastasis. Nomograms, which have received widespread attention in cancer research (3, 28–30), are a simple and effective tool for identifying high-risk individuals and measuring individual risk. No study has yet reported the use of nomograms to predict skip metastases using more detailed clinical data. A previous study developed several prediction models to discriminate patients with skip metastases from those with LLNM, but their clinical applicability was restricted (14, 20, 24, 27).

Due to our ability to identify at-risk skip metastasis patients in the negative CLNM group, we were able to make an informed surgical choice, lessen the likelihood of additional procedures, develop an effective active monitoring plan, and other things. However, our present study has certain drawbacks. First, the current study is a retrospective single-centre investigation. Therefore, its findings can differ slightly from those of other research. To ensure better extrapolation, external validation should be performed, as our nomogram’s validation was only performed internally. Last, there is a lack of long-term monitoring and research on the prognosis of skip metastasis in this study. Despite the fact that our nomogram can identify patients with high-risk skip metastases, it is still unclear whether receiving LLND will increase long-term survival. Therefore, we are conducting an intensive study on the prognosis of skip metastases, such as disease recurrence and postoperative radioactive iodine therapy studies. Notwithstanding these shortcomings, our nomogram is based on good clinical data, has sufficient discriminating power, and has been internally validated in patient populations.

## Conclusion

In conclusion, we created a prediction nomogram for skip metastasis in PTC patients that can assist in identifying patients who require LLND and are at high risk of skip metastasis. Therefore, using this nomogram can help patients make treatment decisions and provide an individual risk assessment.

## Data availability statement

The original contributions presented in the study are included in the article/supplementary material. Further inquiries can be directed to the corresponding authors.

## Ethics statement

The studies involving human participants were reviewed and approved by Ethics Committee of the First Affiliated Hospital of Soochow University. Written informed consent for participation was not required for this study in accordance with the national legislation and the institutional requirements.

## Author contributions

MZ, XS, JC, and BZ conceptualized and designed the study. ZZ, RW, YL, and JL performed analysis. JC and BZ interpreted the data. MZ and XS drafted the manuscript. JC and BZ revised the manuscript. All authors contributed to the article and approved the submitted version.
